# New Year

**Published:** 1850-01

**Authors:** 


*New Year.*—Again we tender to our readers the greeting which custom has appropriated to the commencement of a new year.

To one and all we would say *a happy new year*, not as a phrase of formal courtesy, but with the promptings of a cordial sentiment. Many are the reflections which cluster around this time honored salutation, as we mentally utter it to those whose eyes will scan this page! The mind reverts to the events of the past year, and here, most prominent among the occasions for serious meditation, is the work of the "insatiate archer." Many is the number of those who entered upon the last annual cycle, mayhap full of health, hope and ardor, whom we can no longer greet in this world! On the list selected to go before us, are the names of not a few who were worthily distinguished in the ranks of our profession. Harrison, Hale, Carpenter, Brigham, Barbour, Butterfield, are among the crowd who, within the year that has passed, have been lost to science and humanity. Our thoughts go forward to the commencement of another year, and the solemn truth cannot be repressed, that of those who now interchange greetings which mark the progress of time, all will not remain to participate in their renewal. The moral inference from such a train of reflection requires no homily. Such thoughts, solemn as they are, need not oppress the mind with gloomy apprehensions, and render *New Year's day* a melancholy season. The contemplation of the uncertainty of life, so fitting to the time, in the light of a Christian philosophy, should conduce to that serenity which is the sure and only guaranty of human happiness. Kind readers, if, with the aid of Providence, we may enter on a new probationary period with views and feelings which flow from a true appreciation of the duties and responsibilities of life, whatever may be events of 1850, it will be for us a *happy new year!*

